# Supplementary material for: Economic Burden of SARS-CoV-2 Patients with Multi-Morbidity: A Systematic Review Protocol
Source: Int J Environ Res Public Health. 2022 Oct 13;19(20):13157. doi: 10.3390/ijerph192013157 (PMC9603022; doi:10.3390/ijerph192013157)
Supplement: Supplementary file 1 [file ijerph-19-13157-s001.zip › File S1_DataCollectionForm-Cochrane.pdf]

Suggested citation: Effective Practice and Organisation of Care (EPOC). Data collection form. EPOC Resources for review authors. Oslo: Norwegian Knowledge Centre for the Health Services; 2013. Available at: http://epoc.cochrane.org/epoc-specific-resources-review-authors

THE COCHRANE COLLABORATION®

Data collection form

Intervention review – RCTs and non-RCTs

This form can be used as a guide for developing your own data extraction form. Sections can be expanded and added, and irrelevant sections can be removed. It is difficult to design a single form that meets the needs of all reviews, so it is important to consider carefully the information you need to collect, and design your form accordingly. Information included on this form should be comprehensive, and may be used in the text of your review, 'Characteristics of included studies' table, risk of bias assessment, and statistical analysis.

Notes on using a data extraction form:

- Be consistent in the order and style you use to describe the information for each included study.
- Record any missing information as unclear or not described, to make it clear that the information was not found in the study report(s), not that you forgot to extract it.
- Include any instructions and decision rules on the data collection form, or in an accompanying document. It is important to practice using the form and give training to any other authors using the form.
- You will need to protect the document in order to use the form fields (Tools / Protect document)

**Review title or ID**

**Study ID** *(surname of first author and year first full report of study was published e.g. Smith 2001)*

**Report IDs of other reports of this study** *(e.g. duplicate publications, follow-up studies)*

**Notes:**

1... General Information

1. **Date form completed** *(dd/mm/yyyy)*	
2. **Name/ID of person extracting data**	
3. **Report title** *(title of paper/ abstract/ report that data are extracted from)*	
4. **Report ID** *(if there are multiple reports of this study)*	
5. **Reference details**	
6. **Report author contact details**	
7. **Publication type** *(e.g. full report, abstract, letter)*	

8. **Study funding source** *(including role of funders)*	
**Possible conflicts of interest** *(for study authors)*	
9. **Notes:**	

2... Eligibility

**Study Characteristics**	**Review Inclusion Criteria** *(Insert inclusion criteria for each characteristic as defined in the Protocol)*	**Yes/ No / Unclear**	**Location in text** *(pg & ¶/fig/table)*
10. **Type of study**	Randomised trial	...	
	Non-randomised trial	...	
	Controlled before-after study<br>• Contemporaneous data collection<br>• At least 2 intervention and 2 control clusters	...	
	Interrupted time series OR<br>Repeated measures study<br>• At least 3 timepoints before and 3 after the intervention<br>• Clearly defined intervention point	...<br>...	
	Other design (specify):	...	
11. **Participants**		...	
12. **Types of intervention**		...	
13. **Types of outcome measures**		...	
14. **Decision:**	...		
15. **Reason for exclusion**			
16. **Notes:**			

DO NOT PROCEED IF STUDY EXCLUDED FROM REVIEW

3... Population and setting

	Description<br>*Include comparative information for each group (i.e. intervention and controls) if available*	Location in text<br>*(pg & ¶/fig/table)*
17. **Population description**<br>*(from which study participants are drawn)*		
18. **Setting**<br>*(including location and social context)*		
19. **Inclusion criteria**		
20. **Exclusion criteria**		
21. **Method/s of recruitment of participants**		
22. **Notes:**		

4... Methods

	Descriptions as stated in report/paper	Location in text<br>*(pg & ¶/fig/table)*
23. **Aim of study**		
24. **Design**<br>*(e.g. parallel, crossover, non-RCT)*		
25. **Unit of allocation**<br>*(by individuals, cluster/ groups or body parts)*		
26. **Start date**		
27. **End date**		
28. **Duration of participation**<br>*(from recruitment to last follow-up)*		
29. **Notes:**		

5... Risk of Bias assessment

See Chapter 8 of the Cochrane Handbook. Additional domains may be required for non-randomised studies.

Domain	Risk of bias<br>*Low/ High/Unclear*	Support for judgement	Location in text<br>*(pg & ¶/fig/table)*
30. **Random sequence generation**<br>*(selection bias)*	…		
31. **Allocation concealment**<br>*(selection bias)*	…		

Domain	Risk of bias<br>*Low/ High/Unclear*	Support for judgement	Location in text<br>*(pg & ¶/fig/table)*
32. **Blinding of participants and personnel**<br>*(performance bias)*	…	**Outcome group: All/**	
(if required)	…	**Outcome group:**	
33. **Blinding of outcome assessment**<br>*(detection bias)*	…	**Outcome group: All/**	
(if required)	…	**Outcome group:**	
34. **Incomplete outcome data**<br>*(attrition bias)*	…		
35. **Selective outcome reporting?**<br>*(reporting bias)*	…		
36. **Other bias**	…		
37. **Notes:**			

6… Participants

Provide overall data and, if available, comparative data for each intervention or comparison group.

	Description as stated in report/paper	Location in text<br>(pg & ¶/fig/table)
38. **Total no. randomised**<br>*(or total pop. at start of study for NRCTs)*		
39. **Clusters**<br>*(if applicable, no., type, no. people per cluster)*		
40. **Baseline imbalances**		
41. **Withdrawals and exclusions**<br>*(if not provided below by outcome)*		
42. **Age**		
43. **Sex**		
44. **Race/Ethnicity**		
45. **Severity of illness**		
46. **Co-morbidities**		
47. **Other treatment received**<br>*(additional to study intervention)*		
48. **Other relevant sociodemographics**		
49. **Subgroups measured**		
50. **Subgroups reported**		
51. **Notes:**		

7… Intervention groups

Copy and paste table for each intervention and comparison group

Intervention Group 1

	**Description as stated in report/paper**	**Location in text** *(pg & ¶/fig/table)*
52. **Group name**		
53. **No. randomised to group** *(specify whether no. people or clusters)*		
54. **Description** *(include sufficient detail for replication, e.g. content, dose, components; if it is a natural experiment, describe the pre-intervention)*		
55. **Duration of treatment period**		
56. **Timing** *(e.g. frequency, duration of each episode)*		
57. **Delivery** *(e.g. mechanism, medium, intensity, fidelity)*		
58. **Providers** *(e.g. no., profession, training, ethnicity etc. if relevant)*		
59. **Co-interventions**		
60. **Economic variables** *(i.e. intervention cost, changes in other costs as result of intervention)*		
61. **Resource requirements to replicate intervention** *(e.g. staff numbers, cold chain, equipment)*		
62. **Notes:**		

8… Outcomes

Copy and paste table for each outcome.

Outcome 1

	**Description as stated in report/paper**	**Location in text** *(pg & ¶/fig/table)*
63. **Outcome name**		
64. **Time points measured** *(specify whether from start or end of intervention)*		
65. **Time points reported**		

	Description as stated in report/paper		Location in text *(pg & ¶/fig/table)*
66. **Outcome definition** *(with diagnostic criteria if relevant and note whether the outcome is desirable or undesirable if this is not obvious)*			
67. **Person measuring/ reporting**			
68. **Unit of measurement** *(if relevant)*			
69. **Scales: upper and lower limits** *(indicate whether high or low score is good)*			
70. **Is outcome/tool validated?**	... *Yes/No/Unclear*		
71. **Imputation of missing data** *(e.g. assumptions made for ITT analysis)*			
72. **Assumed risk estimate** *(e.g. baseline or population risk noted in Background)*			
73. **Notes:**			

9... Results

Copy and paste the appropriate table for each outcome, including additional tables for each time point and subgroup as required.

For randomised or non-randomised trial - Dichotomous outcome

	Description as stated in report/paper				Location in text *(pg & ¶/fig/table)*
74. **Comparison**					
75. **Outcome**					
76. **Subgroup**					
77. **Time point** *(specify whether from start or end of intervention)*					
78. **Results** *Note whether:* **...** *post-intervention OR* **...** *change from baseline* *And whether* **...** *Adjusted OR* **...***Unadjusted*	**Intervention**		**Comparison**		
	No. events	No. participants	No. events	No. participants	
79. **Baseline data**	**Intervention**		**Comparison**		
	No. events	No. participants	No. events	No. participants	
80. **No. missing participants and reasons**					

	Description as stated in report/paper		Location in text *(pg & ¶/fig/table)*
81. **No. participants moved from other group and reasons**			
82. **Any other results reported**			
83. **Unit of analysis** *(e.g. by individuals, health professional, practice, hospital, community)*			
84. **Statistical methods used and appropriateness of these methods** *(e.g. adjustment for correlation)*			
85. **Reanalysis required?** *(if yes, specify why, e.g. correlation adjustment)*	... *Yes/No/Unclear*		
86. **Reanalysis possible?**	... *Yes/No/Unclear*		
87. **Reanalysed results**			
88. **Notes:**			

For randomised or non-randomised trial - Continuous outcome

	Description as stated in report/paper						Location in text *(pg & ¶/fig/table)*
89. **Comparison**							
90. **Outcome**							
91. **Subgroup**							
92. **Time point** *(specify whether from start or end of intervention)*							
93. **Post-intervention or change from baseline?**							
94. **Results** *Note whether:* **...** *post-intervention OR* **...** *change from baseline* *And whether* **...** *Adjusted OR* **...***Unadjusted*	**Intervention**			**Comparison**			
	Mean	SD (or other variance)	No. participants	Mean	SD (or other variance)	No. participants	
95. **Baseline data**	**Intervention**			**Comparison**			
	Mean	SD (or other variance)	No. participants	Mean	SD (or other variance)	No. participants	
96. **No. missing participants and reasons**							

	Description as stated in report/paper		Location in text *(pg & ¶/fig/table)*
97. **No. participants moved from other group and reasons**			
98. **Any other results reported**			
99. **Unit of analysis** *(e.g. by individuals, health professional, practice, hospital, community)*			
100. **Statistical methods used and appropriateness of these methods** *(e.g. adjustment for correlation)*			
101. **Reanalysis required?** *(if yes, specify why)*	... *Yes/No/Unclear*		
102. **Reanalysis possible?**	... *Yes/No/Unclear*		
103. **Reanalysed results**			
104. **Notes:**			

For randomised or non-randomised trial - Other outcome

	Description as stated in report/paper				Location in text *(pg & ¶/fig/table)*
105. **Comparison**					
106. **Outcome**					
107. **Subgroup**					
108. **Time point** *(specify whether from start or end of intervention)*					
109. **Type of outcome**					
110. **Results**	Intervention result	SD (or other variance)	Control result	SD (or other variance)	
	Overall results		SE (or other variance)		
111. **No. participant**	Intervention		Control		
112. **No. missing participants and reasons**					
113. **No. participants moved from other group and reasons**					
114. **Any other results reported**					

	Description as stated in report/paper		Location in text *(pg & ¶/fig/table)*
115. **Unit of analysis** *(e.g. by individuals, health professional, practice, hospital, community)*			
116. **Statistical methods used and appropriateness of these methods**			
117. **Reanalysis required?** *(if yes, specify why)*	...		
118. **Reanalysis possible?**	...		
119. **Reanalysed results**			
120. **Notes:**			

For controlled before-after study

	Description as stated in report/paper				Location in text *(pg & ¶/fig/table)*
121. **Comparison**					
122. **Outcome**					
123. **Subgroup**					
124. **Timepoint** *(specify whether from start or end of intervention)*					
125. **Post-intervention or change from baseline?**					
126. **Results**	Intervention result	SD (or other variance)	Control result	SD (or other variance)	
	Overall results		SE (or other variance)		
127. **No. participants**	Intervention		Control		
128. **No. missing participants and reasons**					
129. **No. participants moved from other group and reasons**					
130. **Any other results reported**					
131. **Unit of analysis** *(individuals, cluster/ groups or body parts)*					
132. **Statistical methods used and appropriateness of these methods**					

	Description as stated in report/paper		Location in text *(pg & ¶/fig/table)*
133. **Reanalysis required?** *(specify)*	... *Yes/No/Unclear*		
134. **Reanalysis possible?**	... *Yes/No/Unclear*		
135. **Reanalysed results**			
136. **Notes:**			

For interrupted time series or repeated measures study

	Description as stated in report/paper				Location in text *(pg & ¶/fig/table)*
137. **Comparison**					
138. **Outcome**					
139. **Subgroup**					
140. **Length of timepoints measured** *(e.g. days, months)*					
**Total period measured**					
141. **No. participants measured**					
142. **No. missing participants and reasons**					
143. **No. timepoints measured**	144. **Pre-intervention**		145. **Post-intervention**		
146. **Mean value** *(with variance measure)*					
147. **Difference in means (post – pre)**					
148. **Percent relative change**					
149. **Result reported by authors** *(with variance measure)*					
150. **Unit of analysis** *(individuals or cluster/ groups)*					
151. **Statistical methods used and appropriateness of these methods**					
152. **Reanalysis required?** *(specify)*	... *Yes/No/Unclear*				
153. **Reanalysis possible?**	... *Yes/No/Unclear*				
154. **Individual timepoint results**					
155. **Read from figure?**	... *Yes/No/Unclear*				
156. **Reanalysed results**	**Change in level**	**SE**	**Change in slope**	**SE**	
157. **Notes:**					

10. Applicability

158. **Have important populations been excluded from the study?** *(consider disadvantaged populations, and possible differences in the intervention effect)*	... *Yes/No/Unclear*	
159. **Is the intervention likely to be aimed at disadvantaged groups?** *(e.g. lower socioeconomic groups)*	... *Yes/No/Unclear*	
160. **Does the study directly address the review question?** *(any issues of partial or indirect applicability)*	... *Yes/No/Unclear*	
161. **Notes:**		

11. Other information

	**Description as stated in report/paper**	**Location in text** *(pg & ¶/fig/table)*
162. **Key conclusions of study authors**		
163. **References to other relevant studies**		
164. **Correspondence required for further study information** *(what and from whom)*		
165. **Further study information requested** *(from whom, what and when)*		
166. **Correspondence received** *(from whom, what and when)*		
167. **Notes:**		
