# Supplementary material for: Economic Burden of SARS-CoV-2 Patients with Multi-Morbidity: A Systematic Review Protocol
Source: Int J Environ Res Public Health. 2022 Oct 13;19(20):13157. doi: 10.3390/ijerph192013157 (PMC9603022; doi:10.3390/ijerph192013157)
Supplement: Supplementary file 1 [file ijerph-19-13157-s001.zip › File S2_Article Highlight_IJERPH.pdf]

Article Highlights

Article Title :

Economic Burden of SARS-CoV-2 Patients with Multi-Morbidity: A Systematic Review Protocol

- Primary focus was on the scientific protocol for uncovering economic burden and resources use in Covid 19 pandemic since 2019 to 2021.
- Review recent progress in macro and micro resources utilize combating pandemic for group that suffer pre-existing conditions (comorbidities and multi-morbidities).
- Summarizes the methodology to select quality literature in healthcare system based on conditions mentioned in search strategies.
